# Training International Medical Graduate Internal Medicine Residents in Pelvic Examinations and Pap Smears

**DOI:** 10.1007/s11606-025-09446-1

**Published:** 2025-02-25

**Authors:** Kristin A. Swedish, Nicholas D. Tyau, Nikolina Icitovic, Eva E. Metalios

**Affiliations:** 1 Division of General Internal Medicine, Department of Medicine, Albert Einstein School of Medicine, Montefiore Medical Center, Wakefield Campus, Bronx, NY USA; 2 New York, USA

**Keywords:** international medical graduates, residents, pelvic examinations, pap smears

## Abstract

**Background:**

Nearly 39% of Internal Medicine (IM) residents in American training programs are international medical graduates (IMGs), whose prior training in women’s health is variable. There is a paucity of data about women’s health training programs that train IMG IM residents in pelvic examinations/Pap smears.

**Aim:**

To train IMG IM residents to competently perform pelvic examinations/Pap smears.

**Setting:**

Montefiore Wakefield IM residency clinic, Bronx NY.

**Participants:**

IMG categorical IM residents.

**Program Description:**

In 2015, we established the Women’s Health Clinic (WHC) to train residents in pelvic examinations/Pap smears. The educational model includes one hour of didactics followed by trainees seeing up to four patients, supervised by WHC faculty in 1:2 preceptor to resident ratio.

**Program Evaluation:**

We evaluated the impact of WHC on IMG residents’ self-reported competence in performing pelvic examinations/Pap smears by using McNemar’s test to compare self-reported competence in pelvic examinations and Pap smears before and after WHC participation.

**Discussion:**

WHC improved self-reported competence among IMG residents by utilizing standardized didactics reinforced through immediate and repeated patient interactions. It is an educational model that could be reproduced by other IM programs to improve training for IMG residents.

**Supplementary Information:**

The online version contains supplementary material available at 10.1007/s11606-025-09446-1.

## INTRODUCTION

Although learning to perform pelvic examinations is an integral component of American undergraduate medical education (UME), many Internal Medicine (IM) residents feel ill-prepared. A 2013 study found that 29% of American IM residents reported low confidence in pelvic examinations and Pap smears^1^. Currently, 38.8% of IM residents in American training programs are international medical graduates (IMGs)^2^ who completed UME outside the United States (US). There is no nationwide data specific to IMG IM residents; it is presumed that IMGs similarly do not feel confident in performing pelvic examinations. A needs assessment performed in 2015 in our IMG-predominant IM program found 36% of residents reported low confidence in pelvic exam and 89% did not feel well trained to perform Pap smear (unpublished data).

At the time of our needs assessment, many of our residents were from South Asian countries and reported it was not common for male physicians to perform pelvic examinations due to cultural barriers. Studies from many countries, including the US, report women have negative attitudes towards medical students during obstetric/gynecologic encounters, which can result in fewer medical students being exposed to these exams during UME. Male medical students, in particular, face increased resistance due to cultural and religious attitudes, which may be more common in Muslim countries^3–10^. Acknowledging barriers to training during UME, many IM programs have introduced women’s health curricula combining didactics with clinical exposure to increase exposure and improve confidence^11–15^. A 2019 scoping review identified 16 studies describing innovative curricula in North American IM training programs, only two of which addressed residents’ procedural skills^16^. A 2024 scoping review of obstetrics/gynecology curricula in American IM, family medicine, and pediatrics residency programs identified 81 studies about curricula, only 13 of which assessed procedural skills^17^. None of the studies cited explicitly stated that their training programs included IMG residents (other than our prior study^18^).

There is no literature describing women’s health training programs focusing on teaching IMG IM residents procedural skills. The aim of our innovation was to train IMG IM residents to competently and confidently perform pelvic examinations and Pap smears.

### Setting and Participants

Montefiore Medical Center Wakefield Division is a community hospital located in the Bronx whose 68 categorical IM residents are predominantly IMGs. In 2015, we developed the Women’s Health Clinic (WHC) at our residency clinic to educate and train residents about women’s health and provide needed services for patients.

### Program Description

WHC occurs one afternoon per week and involves two residents supervised by an IM faculty member with experience in women’s health. WHC uses a standardized model with one hour of didactics immediately followed by residents seeing up to four patients, supervised and precepted in 1:2 preceptor to resident ratio. Didactic topics include how to perform pelvic examination/Pap smear presented to all residents during their first session, and then pelvic examination “refresher” provided in conjunction with additional women’s health curricula during subsequent sessions. Following didactics, WHC preceptor accompanied each resident into the exam room and supervised at least their first pelvic exam and Pap smear, assisting and encouraging use of proper techniques. Referral for Pap smear is the most common reason for WHC visit; thus, residents perform multiple pelvic examinations and Pap smears during each WHC session.

As an intervention, WHC itself is not unique; it is similar to other women’s health curricula incorporating didactics and clinical experiences into IM programs to improve procedural skills^11–15^. Its uniqueness lies in its target being IMG residents.

### Program Evaluation

In 2015, we evaluated residents’ experiences following WHC participation. Our study found that one WHC session increased residents’ self-reported competence and confidence in providing women’s healthcare.^18^ However, that study did not evaluate residents’ pre-US educational experiences, and we were unable to determine if improvements were primarily due to new acquisition of procedural skills.

We designed the current study to better assess the impact of WHC on IMG residents’ self-reported competence in performing pelvic examinations and Pap smears by evaluating their prior non-US training in women’s health. In spring 2023, all 68 categorical IM residents were sent Google Survey questionnaire with informed consent. In July 2024, all 22 PGY2 residents, who were not residents at the time of first survey administration, were also sent the survey. This protocol was approved by the Albert Einstein Human Research Protection Program as exempt.

The survey included demographics, questions about trainees’ experiences learning pelvic examinations during non-US UME and non-US graduate medical education (GME), and how many pelvic examinations performed prior to Wakefield IM residency. The survey also included four self-assessment questions relating to specific competencies on pelvic examinations and Pap smears using a 5-point Likert scale (strongly agree, agree, neutral, disagree, disagree strongly). Participants were asked to assess competencies both prior to and following WHC participation in the same survey. Surveys of participants who had not yet participated in WHC bypassed post-WHC questions. Survey questions were adapted from a prior intervention that introduced pelvic examination training into an IM program.^11^ Our survey also asked residents to identify when they first felt comfortable and confident performing pelvic examinations, if they had encountered any barriers during WHC, and if they had recommendations for improvement (Appendix).

Survey results were imported into IBM SPSS Statistics Version 29.0, which provided counts and percentages. Fisher’s exact test was used to compare data of those who had and had not participated in WHC. For those who had participated in WHC, their responses to the four self-assessment questions before and after WHC were compared using McNemar’s test. Likert scale data was dichotomized with agree and strongly agree indicating competence and strongly disagree, disagree, and neutral signifying lack of competence. Fisher’s exact test was also used to estimate the dose–response effect of more than one WHC session on self-assessment questions.

Sixty of 90 IM residents completed the survey, yielding 67% response rate. The one US-UME trained resident was removed so all 59 respondents were IMGs, representing nine world regions. All three post-graduate years (PGY) were equally represented. Survey respondents were 53% male and 47% female, representative of overall gender makeup of the program. Fifty-six residents (95%) reported learning to perform pelvic examinations during non-US UME training. Seventy-four percent of those who completed non-US GME training reported learning pelvic examinations during GME. Sixty-three percent and 58% reported learning how to perform Pap smears during prior UME and GME training, respectively. Prior to US IM residency, although 20% reported never performing a pelvic examination, 37% reported 1–4 prior, 10% reported 5–9 prior, and 32% reported more than 10 prior pelvic examinations.

Forty-nine residents (83%) participated in WHC, 33 (56%) of whom attended more than one session in the prior year (median 2–3 sessions). Compared to those who had not participated in WHC, those who participated in WHC were more likely to be PGY 2/3 (*p* = 0.002) and to have performed more pelvic exams in the past (*p* = 0.019). Gender, age, marital status, and whether they were taught pelvic examinations and Pap smears during prior UME or GME were comparable between both groups (Table 1). When asked when they first felt comfortable and confident in performing pelvic examinations, more residents reported feeling both comfortable and confident following WHC than either following UME or GME (45% and 41%, respectively, compared to 27% and 16% UME and 25% and 25% GME).

**Table 1:** Demographics and Prior Experiences of Those Who Had and Had Not Participated in WHC

	WHC Participation (*N* = 49) *N* (%)	No WHC Participation (*N* = 10) *N* (%)	*p*-value
PGY Year			
PGY1	8 (16)	7 (70)	0.002
PGY2	26 (54)	3 (30)	
PGY3	15 (30)	0	
Female	25 (51)	3 (30)	0.306
Age			
25–29 years old	18 (37)	5 (50)	0.508
30–34 years old	28 (57)	4 (40)	
35–39 years old	2 (4)	1 (10)	
40 + years old	1 (2)	0	
Marital Status			
Never married	27 (55)	5 (50)	0.784
Married	21 (43)	5 (50)	
Separated/divorced	1 (2)	0	
Number of Prior Pelvic Exams			
0 prior pelvic exams	8 (16)	4 (40)	0.019
1–4 prior pelvic exams	16 (33)	6 (60)	
5–9 prior pelvic exams	6 (12)	0	
10 + prior pelvic exams	19 (39)	0	
Learned pelvic during UME	48 (98)	8 (80)	0.072
Learned Pap during UME	32 (67)	3 (38)	0.136
Learned pelvic during GME	26 (77)	5 (63)	0.412
Learned Pap during GME	16 (61.5)	2 (40)	0.625

Residents who participated in WHC (*n* = 49) assessed themselves as more competent on all four competence questions as compared to prior to their WHC participation. Of note, 62.5% of residents who felt unable to competently perform pelvic examinations prior to WHC reported being able to competently perform pelvic examinations following WHC (*p* = 0.002). More than 70% of residents who were unable to locate the cervix prior to WHC were able to locate the cervix following WHC (*p* < 0.001). Eighty-eight percent of residents unable to obtain an adequate Pap smear sample as per pathology prior to WHC reported being able to obtain an adequate sample following WHC (*p* < 0.001). Despite an increase in self-reported competence, only 42.4% of those who reported not being likely to perform routine pelvic examinations prior to WHC stated they would be likely to perform pelvic examinations following WHC (*p* < 0.001; Table 2). A significant dose-dependent effect was found: residents who participated in two or more WHC sessions were more likely to feel competent in all three clinical skills than those who participated only once (competently perform pelvic *p* = 0.025, locate cervix *p* = 0.030, perform adequate Pap *p* = 0.030). However, they were not significantly more likely to perform routine pelvic exams (*p* = 0.215).

**Table 2:** Self-assessed Competence Before and After WHC

	No *N* (%)	Yes *N* (%)	*p*-value
**After WHC: I am able to competently perform pelvic examinations**	**Before WHC: I was able to competently perform pelvic examinations**		0.002
No	9 (37.5)	2 (8)	
Yes	15 (62.5)	23 (92)	
Total	24	25	
**After WHC: When performing a speculum exam, I am able to locate the cervix**	**Before WHC: When performing a speculum exam, I was able to locate the cervix**		< 0.001
No	7 (29.2)	0	
Yes	17 (70.8)	25 (100)	
Total	24	25	
**After WHC: I am able to obtain an adequate sample (as per pathology report) when performing a Pap smear**	**Before WHC: I was able to obtain an adequate sample (as per pathology report) when performing a Pap smear**		< 0.001
No	3 (12)	0	
Yes	22 (88)	24 (100)	
Total	25	24	
**After WHC: I am likely to perform routine pelvic examinations on my female patients**	**Before WHC: I was likely to perform routine pelvic examinations on my female patients**		< 0.001
No	19 (57.6)	1 (6.3)	
Yes	14 (42.4)	15 (93.8)	
Total	33	16	

Although most residents noted no barriers in WHC experience, gender was reported as a barrier by nine residents (18%). “Some female patients prefer female providers,” one resident stated. Residents recommended improving WHC by incorporating the use of pelvic exam models/mannequins (20 residents (41%)) and standardized patients (8 residents (16%)).

## DISCUSSION

This study has several limitations. First, it documents the particular experiences of IMG IM residents at Montefiore Wakefield, which may not generalize to other residency programs with IMG trainees. However, our residents represent nearly all world regions, providing diversity in background and training experiences that may truly yield a representative sample. Second, our survey response rate was 67%. Residents who chose not to complete the survey may have had different experiences that are not represented in current findings. However, there were no demographic differences between survey responders and non-responders. Third, the same survey asked about participants’ self-rated competence both prior to and after WHC, which could have been impacted by recall bias. Fourth, although many questions were asked to evaluate residents’ experiences in UME/GME training prior to coming to Wakefield, survey may not have fully assessed all factors that contribute to competence in performing pelvic examinations and Pap smears. Fifth, follow-up survey of residents reassessing their competence would be helpful to determine if WHC impact is sustained over time. Finally, we were unable to incorporate objective measures of competence and skill in pelvic examinations and Pap smears, instead relying on resident self-report.

Our prior study found one session in WHC increased IM residents’ comfort and competence in pelvic examinations, a result we considered possibly skewed given trainees were IMGs who might have had less training in women’s health prior to US residency. This study, however, found that most of our IMG residents had been taught how to perform pelvic examinations during prior non-US UME/GME training, and many had performed a significant number of pelvic examinations previously. Similar to US-trained IM residents, prior education about women’s health did not improve confidence in procedures among our IMG residents. Participation in WHC improved residents’ self-reported competence in their ability to perform pelvic examinations competently, their ability to locate the cervix, and their ability to obtain an adequate pathology sample when performing a Pap smear. Unfortunately, despite an increase in self-assessed competence, the majority of residents continued to note being unlikely to perform routine pelvic examinations on their female patients. This may result from the gender barrier noted by a small number of residents, or other common barriers to incorporating pelvic examinations into routine primary care, such as time.

We believe that the WHC educational model of dedicated time for standardized basic didactics followed by immediate and repeated supervised patient interactions creates an environment that normalizes learning pelvic examinations while providing trainees time to focus on acquiring the specific skill set. This combination of didactics followed by guided practice has been found to work well in teaching sensitive exams, including pelvic examinations, while also minimizing learner anxiety and fear, similar to other women’s health curricula successfully administered to American IM residents^11–15,19^. However, no prior published innovations have described successful educational models that teach pelvic examination skills specifically to IMG IM residents.

WHC is a procedural skills training program that has shown to increase IMG trainee competence in performing pelvic examinations and Pap smears despite prior training in non-US UME/GME. As such, it is a model that could be utilized by other predominantly IMG IM residency programs to ensure that IMG trainees develop confidence in pelvic examination skills. WHC utilizes a general internist preceptor and requires no specialized equipment, making it feasible for implementation by other programs.

## Supplementary Information

Below is the link to the electronic supplementary material.

Supplementary file1 (DOCX 24 KB)

## Data Availability

Data is available upon request.
