# Supplementary material for: Training International Medical Graduate Internal Medicine Residents in Pelvic Examinations and Pap Smears
Source: J Gen Intern Med. 2025 Feb 25;40(9):2075–9. doi: 10.1007/s11606-025-09446-1 (PMC12325812; doi:10.1007/s11606-025-09446-1)
Supplement: Supplementary file 1 — Supplementary file1 (DOCX 24 KB) [file 11606_2025_9446_MOESM1_ESM.docx]

INFORMED CONSENT

You are being asked to participate in a research study entitled "Exploring Wakefield Internal Medicine Residents’ Background in Women’s Health in Order to Improve Training in Women’s Health Care via the Women’s Health Clinic at Montefiore Wakefield Ambulatory Care Center: A Questionnaire Study" because you are an internal medicine resident at Wakefield who has or will participate in the Women’s Health Clinic. The purpose of this study is to determine how your prior experiences impact your experience in the Women’s Health Clinic. The results of this survey will help optimize how we teach about women’s health, including pelvic examination and Pap smears. Below you will find the survey which will take 10-15 minutes to complete. Thank you for taking the time to complete this survey!

Participation is voluntary. All data collected in this survey are *confidential*. You may skip any question you do not wish to answer and are under no obligation to complete the survey.

Participation in this survey is completely voluntary. The results of this study will be disseminated in a de-identified aggregate manner. If you have questions, you can call Dr. Kristin Swedish at 347-341-4300 or kswedish@montefiore.org. You can call and ask questions at any time.

1. In what region did you perform the majority of your undergraduate medical education?
   - Caribbean
   - Central and South America
   - Eastern, Western, and Southern Africa
   - Europe (including Russia and the former USSR)
   - Middle East and North Africa (Algeria, Bahrain, Cyprus, Egypt, Iran, Iraq, Israel, Jordan, Kuwait, Lebanon, Libya, Morocco, Oman, Palestine, Qatar, Saudi Arabia, Syria, Tunisia, Turkey, United Arab Emirates, Yemen)
   - North America (Canada, Mexico; not US)
   - South Asia (Bangladesh, Bhutan, India, Maldives, Nepal, Pakistan, Sri Lanka, Afghanistan)
   - Southeast Asia (Brunei, Cambodia, Indonesia, Laos, Malaysia, Phillipines, Singapore, Thailand, Timor-Leste, Vietnam)
   - East Asia (China, Hong Kong, Japan, Macao, Mongolia, North Korea, South Korea, Tibet)
   - United States
2. Were you taught how to perform a pelvic examination during undergraduate medical education?
   - Yes
   - No
3. If Yes to Question 2, were you taught how to perform a pelvic examination during preclinical course work or during clinical clerkships?
   - Preclinical course work
   - Clinical clerkship
4. If Yes to Question 2, what method was used when you were taught how to perform a pelvic examination during undergraduate medical education? (select all that apply)
   - Pelvic exam models/mannequins
   - Standardized patients (gynecologic teaching assistants)
   - Observed clinical encounters (watching other clinicians perform pelvic exam)
   - Participated in clinical encounters (you performed pelvic exam yourself)
   - Other: please explain
5. If Yes to Question 2, were you also taught how to perform a Pap (or cervical) smear when you learned how to perform a pelvic examination during your undergraduate medical education?
   - Yes
   - No
6. If No to Question 2, why were you not taught the pelvic examination during your undergraduate medical education? (free text)
7. Did you perform an internship/residency in your home country or another country prior to coming to the United States?
   - Yes
   - No
8. If Yes to Question 7, in what region did you perform the majority of your internship/residency?
   - Caribbean
   - Central and South America
   - Eastern, Western, and Southern Africa
   - Europe (including Russia and the former USSR)
   - Middle East and North Africa (Algeria, Bahrain, Cyprus, Egypt, Iran, Iraq, Israel, Jordan, Kuwait, Lebanon, Libya, Morocco, Oman, Palestine, Qatar, Saudi Arabia, Syria, Tunisia, Turkey, United Arab Emerites, Yemen)
   - North America (Canada, Mexico; not US)
   - South Asia (Bangladesh, Bhutan, India, Maldives, Nepal, Pakistan, Sri Lanka, Afghanistan)
   - Southeast Asia (Brunei, Cambodia, Indonesia, Laos, Malaysia, Phillipines, Singapore, Thailand, Timor-Leste, Vietnam)
   - East Asia (China, Hong Kong, Japan, Macao, Mongolia, North Korea, South Korea, Tibet)
   - United States
9. If Yes to Question 7, were you taught how to perform a pelvic examination during that internship/residency?
   - Yes
   - No
10. If Yes to Question 7, how were you taught to perform a pelvic examination during internship/residency?
    - Pelvic exam models/mannequins
    - Standardized patients (gynecologic teaching assistants)
    - Observed clinical encounters (watching other clinicians perform pelvic exam)
    - Participated in clinical encounters (you performed pelvic exam yourself)
    - Other (please explain)
11. If Yes to Question 7, were you also taught how to perform a Pap (or cervical) smear when you learned how to perform a pelvic examination?
    - Yes
    - No
12. If No to Question 7, why did you not learn the pelvic examination during your internship/residency? (free text)
13. Did you encounter any of the following barriers to while learning how to perform a pelvic examination during either your undergraduate medical education or your internship/residency? (select all that apply)
    - Gender
    - Religion
    - Marital status
    - Sexual orientation
    - Other: (please explain)
    - None
    - Free text
14. Prior to Montefiore Wakefield IM residency, how many pelvic exams had you performed:

- 0
- 1-4
- 5-9
- ≥ 10

1. To what extent do you agree or disagree with the following statements about your

experience performing pelvic examinations **PRIOR TO** starting Montefiore Wakefield IM residency? (Likert scale: strongly agree, agree, neutral, disagree, disagree strongly)

- I was able to competently perform pelvic examinations
- When performing a speculum exam, I was able to locate the cervix
- I was able to obtain an adequate sample (as per pathology report) when performing a Pap smear
- I was likely to perform routine pelvic examinations on my female patients

1. Regardless of your prior experiences with pelvic examinations during undergraduate medical education and/or internship/residency, how would you prefer to be taught the pelvic examination at Montefiore Wakefield? (select all that apply)
   - Pelvic exam models/mannequins
   - Standardized patients (gynecologic teaching assistants)
   - Observed clinical encounters (watching other clinicians perform pelvic exam)
   - Participate in clinical encounters (you perform pelvic exam yourself)
   - Other (please explain)
2. Have you participated in the WHC at any time during your residency?
   - Yes
   - No

If Yes to Question 17, please complete Questions 18-24. If No to Question 17, then this survey is nearly complete (skip to #25 for Demographic data)

1. If Yes to Question 17, how many times have you participated in the WHC during residency?

- 0
- 1
- 2-3
- 4-6
- 7+

1. What has been helpful about the WHC experience in terms of improving your comfort with and confidence in learning the pelvic examination? (free text)
2. What has been not helpful about the WHC experience in terms of improving your comfort with and confidence in learning the pelvic examination? (free text)
3. Have you encountered any of the following barriers to while performing pelvic examinations at the WHC? (select all that apply)
   - Gender
   - Religion
   - Marital status
   - Sexual orientation
   - Other: (please explain)
   - None
   - Free text
4. When did you first feel **comfortable** performing pelvic examinations?

- During medical school
- During prior internship/residency
- During Wakefield IM residency
- Do not yet feel comfortable performing pelvic examinations

1. When did you first feel **confident in** performing pelvic examinations?

- During medical school
- During prior internship/residency
- During Wakefield IM residency
- Do not yet feel confident performing pelvic examinations

1. To what extent do you agree or disagree with the following statements about your

experience performing pelvic examinations **NOW**? (Likert scale: strongly agree, agree, neutral, disagree, disagree strongly)

- I am able to competently perform pelvic examinations
- When performing a speculum exam, I am able to locate the cervix
- I am able to obtain an adequate sample (as per pathology report) when performing a Pap smear
- I am likely to perform routine pelvic examinations on my female patients

1. What year are you?

- PGY1
- PGY2
- PGY3
- PGY4

1. What is your gender identity?

- Female
- Male
- Other

1. How old are you?

- 20-24
- 25-29
- 30-34
- 35-39
- 40-44
- 45+

1. What is your marital status?

- Never married
- Married
- Separated/Divorced/Widowed
